# Comparisons and Refinements of Neonatal Oro-Tracheal Intubation Length Estimation Methods in Taiwanese Neonates

**DOI:** 10.3389/fped.2020.00367

**Published:** 2020-07-10

**Authors:** Chun-Chih Peng, Hung-Yang Chang, Ngiik-Ping Tiong, Jui-Hsing Chang, Chyong-Hsin Hsu, Wai-Tim Jim, Chia-Ying Lin, Chia-Hui Chen, Mary Hsin-Ju Ko

**Affiliations:** ^1^Department of Pediatrics, MacKay Children's Hospital, Taipei, Taiwan; ^2^Department of Medicine, MacKay Medical College, New Taipei City, Taiwan; ^3^Department of Pediatrics, Hsinchu MacKay Memorial Hospital, Hsinchu, Taiwan

**Keywords:** newborn, preterm, resuscitation, intubation, trachea

## Abstract

**Objective:** This study aimed to evaluate the efficacy of Tochen's formula [TF, body weight (kg) plus 6 cm], nasal septum to ear tragus length (NTL) + 1 cm, and Neonatal Resuscitation Program gestational age (NRP-GA) and body weight (NRP-BW)-based intubation table in estimating the oro-tracheal intubation length, and to improve the estimation efficacy using anthropometric measurements in Taiwanese neonates.

**Study design:** This was a prospective observational study conducted at a neonatal intensive care unit in Taipei, Taiwan. One hundred intubated neonates were enrolled. The estimated intubation depth was defined as being mid-tracheal concordant if it placed the endotracheal tip between the upper border of the first and the lower border of the second thoracic vertebra. A linear regression model was used to analyze the relationships between mid-tracheal depth and body weight (BW), NTL and gestational age (GA), and to revise the NRP intubation tables using our results.

**Results:** Overall, 56% of the neonates were born at a GA ≤ 28 weeks and 48% had a BW ≤ 1,000 g. The overall mid-tracheal concordance rates for TF, NTL + 1 cm, NRP-GA, and NRP-BW estimations were 51.0, 57.0, 15.0, and 14.0%, and in the infants with a BW ≤ 1,000 g 56.3, 56.3, 8.3, and 8.3%, respectively. Our revisions of the NRP intubation tables based on the anthropometric measurements of our participants improved the efficacy of BW, GA, and NTL estimations to 63, 44, and 61%, respectively.

**Conclusion:** TF and NTL + 1 cm were more reliable than NRP intubation tables in predicting the neonatal mid-tracheal length in neonates of all BW and GA. Considering morphological differences secondary to ethnicity, we recommend using these tailored recommendations during neonatal resuscitation in Asian neonates.

## Introduction

Establishing an effective artificial airway is pivotal to neonatal resuscitation. However, nearly one in four intubation attempts result in endotracheal tube tip (ETT) malpositioning (1). Given that the vocal cords and carina are in close proximity, neonates, and especially those born prematurely, are prone to pronounced ETT displacements with slight neck movements (2). Effective ETT placement is crucial to initiate resuscitation timely, expedite patient stabilization, and avoid complications, including oxygen desaturation, air leak syndrome, atelectasis and pulmonary hemorrhage (3–6).

The ETT should ideally be positioned within the mid-tracheal region (7). However, optimal placement may be affected by point of measurement references, neck position and respiratory phase, making an accurate determination of ETT position clinically challenging. Although the time to successful intubation may be as short as 30 s, the time to radiological confirmation of ETT position is usually much longer and may not always be feasible prior to emergency procedures, such as surfactant administration or inter-hospital neonatal transport (1, 8).

Various methodologies to estimate the ideal position and definitions of optimal placement of the ETT have been proposed. The Neonatal Resuscitation Program (NRP) previously recommended using Tochen's formula (TF) (body weight in kg plus 6 cm) to estimate the oro-tracheal distance (OTD) (9). However, in the 7th edition of the NRP, TF was replaced by body weight (BW) and gestational age (GA)-based intubation tables, and the nasal septum to ear tragus length (NTL) + 1 cm formula (3, 7, 10). Because the estimation methods were developed based on Caucasian measurements, the efficacy of these methods in non-Caucasian neonates needs to be validated. The aim of this study was to compare these OTD estimation methods in Asian neonates, and especially those who were extremely preterm (EP) and those who had an extremely low birth weight (ELBW).

## Methods

This was a prospective observational study conducted at a single neonatal intensive care unit (NICU) at MacKay Children's Hospital in Taipei, Taiwan from May 2017 to December 2018. The study was approved by the Institutional Review Board of Mackay Memorial Hospital (IRB 17MMHIS035e) and parental consent was obtained before enrollment.

Only infants intubated within 24 h after birth were recruited to minimize the potential influence of physiological post-natal dehydration on morphological measurements. Infants with dysmorphic facial features, airway and lung anomalies, and those without NTL measurements were excluded. For patients requiring invasive ventilation, the standard of care in our unit was to intubate orally using a weight-appropriate endotracheal tube (Mallinckrodt™ tracheal tube, Covidien, Mansfield, USA) and straight laryngoscope blades.

A portable antero-posterior CXR with digital measurements was obtained after endotracheal intubation. Prior to chest imaging, as per NICU protocol, the endotracheal tube was secured at the mid-upper lip, and the head was positioned in a neutral position with a gel positioner and shoulder roll. The radiographs were taken by radiologic technicians on the first day of life, using a mobile Shimadzu MobileArt Evolution MX7 (Shimadzu Corporation, Kyoto, Japan), and Fujiflm IP Cassette Type CC film (Fujifilm Corporation, Tokyo, Japan). All radiographs were processed electronically, with a physical scale of 1:1.

**Figure 1:**
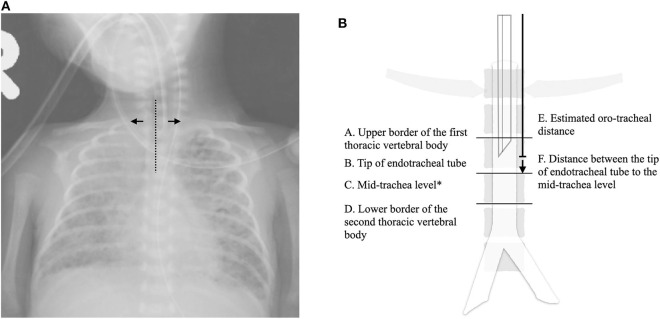
Illustrations of the radiological features interpreted on chest roentgenograms. **(A)** An example of a good quality chest roentgenogram qualified for further interpretations: trachea in the midline (dotted line) and clavicles horizontally positioned (arrow). **(B)** A schematic diagram of the radiological features of interest. The correct oro-tracheal distance was derived from E + F. *C = (A + D)/2. **Table:** 

The CXRs were reviewed independently by two neonatologists (NT and MK) who were blinded to the depths of insertion. NT and MK were responsible for determining the quality of the CXR, and to delineate radiological landmarks and position of the ETT on the CXR. Only CXRs with the trachea in the midline and clavicles horizontally positioned were qualified for interpretation (Figure 1). The mid-trachea, spanning from the upper margin of the first vertebral body and the lower margin of the second vertebral body (T1T2), was considered to be the optimal ETT position on CXR (7). The OTD was defined as the length of orally intubated ETT measured from the mouth to mid-trachea (11). We estimated the OTD using the following four methods: TF, NTL, and the NRP recommendations of gestational age (NRP-GA) and weight (NRP-BW)-based intubation lengths, and compared their (1) accuracy and (2) concordance to the measured OTDs. If the estimated depth placed the ETT between T1T2, the results were defined as being mid-tracheal concordant. If the ETT was placed above the upper margin of T1, the estimated depth was defined as being too shallow. Conversely, if the ETT was below the lower margin of T2, the estimated depth was defined as being too deep. The distance from the ETT to mid-trachea level and the correct OTD were calculated from radiological features identified on CXR (Figure 1B). The growth chart of the Taiwanese population published by Hsieh et al. was used as the growth standard, and small for gestational age (SGA) was defined as infants weighing below the 10th percentile (12). Extremely preterm (EP) infants were defined as those delivered at a GA ≤ 28 weeks, and extremely low birth weight (ELBW) infants as those born with a BW ≤ 1,000 g.

**Symbol**	**Explanation**
**(A)**	
— — —-	Tracheal alignment
→	Clavicular alignment
**(B)**	
A	Upper border of first thoracic vertebral body
B	Tip of endotracheal tube
C	Mid-trachea level
D	Lower border of second thoracic vertebral body
E	Estimated oro-tracheal distance
F	Distance between the endotracheal tip and the mid-trachea

### Statistics

Measured OTD was calculated as the distance from the ETT to the mid-point between T1T2 plus the actual intubated length. Estimated OTDs were derived from the estimation methods. The overall mid-tracheal concordance rates of the different methods were compared using the chi-square test, and mean OTDs were compared using independent Student's *t*-tests. Regression equations from NTL, BW, and GA were calculated to estimate OTDs, or the predicted intubation length from the mid-upper lip to the midpoint of the mid-trachea (tip-to-lip). All statistical analyses were performed using SPSS v25.0 (SPSS Inc, Chicago, IL). A *p*-value of <0.05 was considered to be statistically significant.

## Results

**Figure 2:**
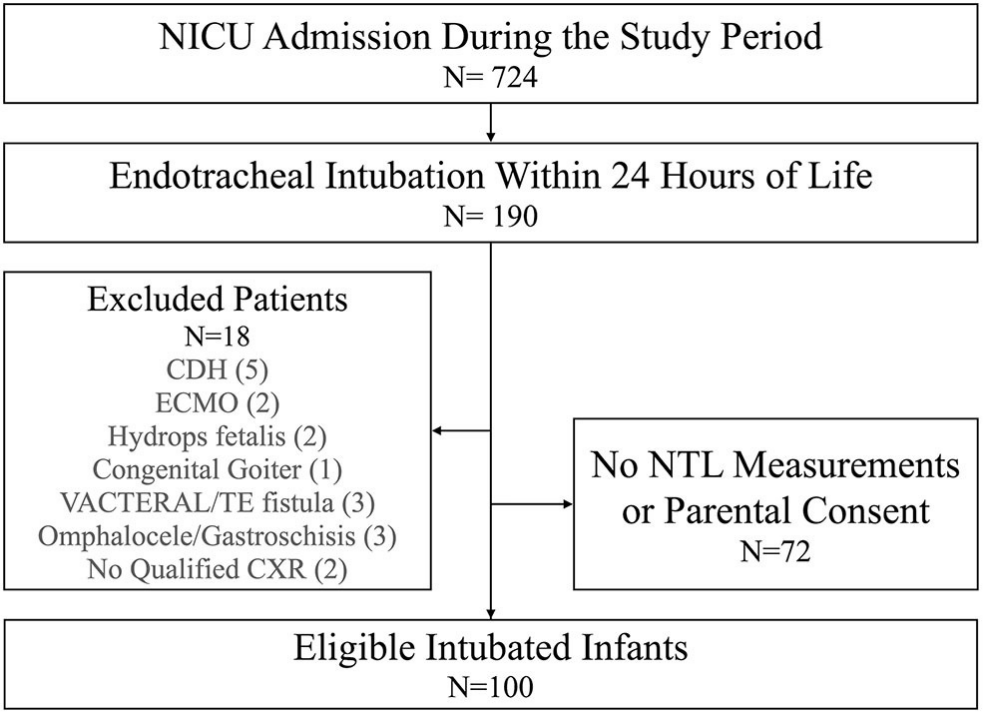
Flow chart describing patient inclusion and exclusion. CDH, congenital diaphragmatic hernia; CXR, chest X ray; ECMO, extracorporeal membrane oxygenation; NICU, neonatal intensive care unit; NTL, nasal septum to ear tragus length; TE fistula, trachea-esophageal fistula; VACTERAL, vertebral anomaly, cardiac anomaly, TE fistula, esophageal atresia, renal/radial anomalies, limb defects.

During the study period, there were 724 admissions to our unit, and 190 infants were intubated within 24 h of life (Table 1). After excluding three neonates with congenital anomalies, three neonates with dysmorphic facial features, four neonates with no eligible CXRs, and neonates without parental consent, a total of 100 infants were enrolled (Figure 2). Of these infants, 78 (78.0%) were preterm, 86 (86.0%) had a BW < 2,500 g, 25 (25.0%) were SGA, and 57 (57.0%) were male. The average OTD was 7.5 ± 1.1 cm, and the average distance between T1 and T2 was 0.8 ± 0.2 cm.

**Table 1:** Patient demographics.

	***n***
Total (%)	100
Male (%)	57
LGA (%)	7
SGA (%)	25
BW ± SD (g)	1389.9 ± 855.5
BW ≤ 1,000 (%)	48
BW 1,001–1,500 (%)	20
BW 1,501–2,500 (%)	18
BW > 2,500 (%)	14
GA ± SD (week)	29.7 ± 5.3
GA ≤ 26 (%)	42
GA 27–32 (%)	32
GA 33–36 (%)	4
GA > 37 (%)	22

*BW, Body Weight; GA, Gestational Age; LGA, Large for Gestational Age; SD, Standard Deviation; SGA, Small for Gestational Age*.

### Accuracy of the Estimation Modalities

**Figure 3:**
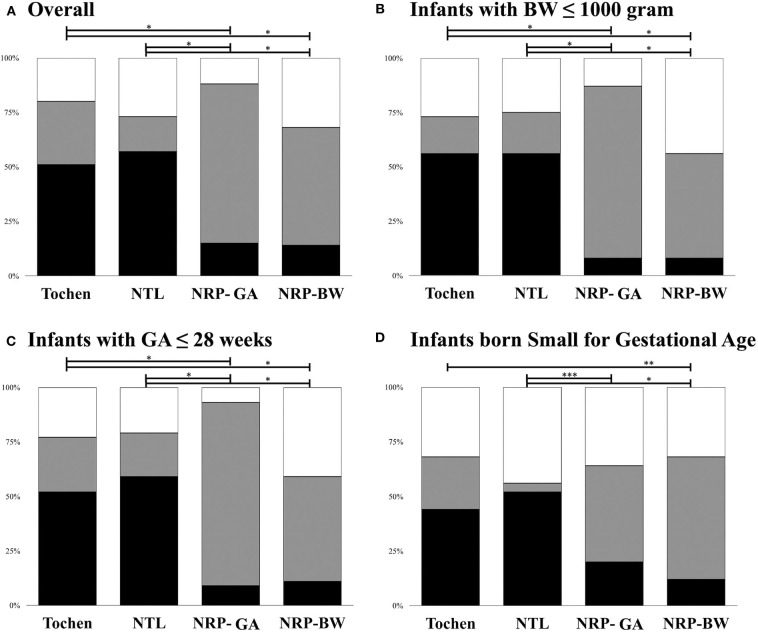
Positioning of the endotracheal tube tip in relation to the mid-trachea using TF, NTL, and NRP intubation length recommendations. **(A)** For all participants; **(B)** For infants with very low birth weight; **(C)** For infants born before completion of 28 weeks of gestational; **(D)** For infants born small for gestational age. Black: concordant with mid-trachea. Gray: shallower than mid-trachea. White: deeper than mid-trachea. TF, body weight + 6 cm; NTL, Nasal Tragus Length + 1 cm; NRP, Neonatal Resuscitation Program; NRP-GA, NRP Intubation Length Recommendation by Gestational Age; NRP-BW, NRP Intubation Length. **Table:** 

The average results of TF were most compatible with the OTDs (*p* = 0.43) (Table 2). The OTDs were overestimated by NTL and underestimated by NRP-GA and NRP-BW. Nonetheless, TF and NTL + 1 cm had an overall comparable mid-tracheal concordance (TF: 51.0%, NTL: 57.0%, *p* = 0.48) (Figure 3). NRP-BW and NRP-GA also had comparable mid-tracheal concordance (NRP-BW 14.0%, NRP-GA 15.0%), and were both significantly lower than those of TF and NTL.

**Table 2:** Comparisons of actual oro-tracheal distance to the results of different estimation methodologies.

	***n***	**Oro-tracheal distance ± SD (cm)**	**Intubation Length** **±** **SD predicted by (cm)**			
			**Tochen**	**NTL**	**NRP-GA**	**NRP-BW**
Total	100	7.4 ± 1.1	7.4 ± 0.9	7.5 ± 1.1^*^	6.8 ± 1.1^*^	7.1 ± 1.2^*^
ELBW (%)	48	6.6 ± 0.5	6.8 ± 0.1	6.7 ± 0.4	6.0 ± 0.4^*^	6.9 ± 1.3
EP (%)	56	6.8 ± 0.6	6.8 ± 0.2	6.8 ± 0.5	6.0 ± 0.4^*^	6.9 ± 1.3
SGA (%)	25	7.4 ± 1.0	7.3 ± 0.7	7.7 ± 1.1^*^	7.3 ± 1.1	7.3 ± 1.2

*BW, Body Weight; ELBW, Extremely Low Birth Weight; EP, Extremely Preterm; GA, Gestational Age; NRP-GA, Neonatal Resuscitation Program Intubation Length Recommendation by Gestational Age; NRP-BW, Neonatal Resuscitation Program Intubation Length Recommendation by Body Weight; NTL, Nasal Tragus Length + 1 cm; SD, Standard Deviation; SGA, Small for Gestational Age; Tochen, Body Weight + 6 cm*.

* *Significantly differed from the oro-tracheal distance, p < 0.05*.

**Shading**	**Explanation**
■	Concordant with mid-trachea
■	Shallower than mid-trachea
□	Deeper than mid-trachea

### Efficacy by Body Weight and Gestational Age

Estimates of TF and NRP-BW were too shallow in the younger infants but too deep in the older infants by different degrees. NTL estimations were deeper than measured OTDs in the younger infants and shallower in the older infants. NRP-GA estimates were invariably shallower than measured OTDs irrespective of BW.

#### Extremely Low Birth Weight Infants

Among the 48 ELBW infants, the measured OTD was 6.6 ± 0.5 cm, and the mid-trachea spanned 0.7 ± 0.1 cm (Figure 3). TF had better mid-tracheal concordance in the ELBW infants (ELBW: 56.3% vs. non-ELBW 46.2%, *p* = 0.02). NTL estimates also had similar concordance (ELBW: 56.3% vs. non-ELBW 52.6%, *p* = 0.75). While the overall concordance of NRP-GA did not differ significantly between the ELBW and non-ELBW infants (ELBW 8.3% vs. non-ELBW 19.2%, *p* = 0.20), NRP-BW was more likely to result in intubations that were too deep (ELBW: 43.8% vs. non-ELBW 21.2%, *p* = 0.04).

#### Extremely Preterm Infants

The measured OTD was 6.8 ± 0.6 and the mid-trachea spanned 0.7 ± 0.1 cm in the EP infants. Both TF (EP: 51.8%, vs. non-EP: 50.0%, *p* = 0.50) and NTL (EP: 58.9%, vs. non-EP: 54.5%, *p* = 0.37) had better mid-tracheal concordance in the EP neonates. Although the concordance of NRP-BW was only modestly lower in the EP infants (EP: 10.7%, vs. non-EP: 18.2%, *p* = 0.08), NRP-GA estimates had a significantly lower mid-tracheal concordance rate (EP: 8.9%, vs. non-EP: 22.7%, *p* = 0.02*)* (Figure 3).

#### Small for Gestational Age Infants

The measured OTD was 7.4 ± 1.0 cm and the mid-trachea spanned 0.8 ± 0.2 cm in the SGA infants. While the mid-tracheal concordance rate of NTL estimates was significantly lower in the SGA infants (SGA: 52.0% vs. non-SGA 58.7%, *p* = 0.04), NRP-GA had better efficacy (SGA: 20.0% vs. non-SGA 13.3%, *p* < 0.001). In contrast, the efficacy of weight-based estimates, including TF (*p* = 0.22) and NRP-BW (*p* = 0.94), exhibited only minor changes among the SGA infants.

### Modifications to the Current Modalities

Measured OTDs and BW, GA, and NTL were positively correlated (Table 3). The results derived from the different parameters were simplified by rounding to the nearest half centimeter and applied to our participants (Table 4). The mid-tracheal concordance rates of the simplified results were 63, 44, and 61% using BW, GA, and NTL, respectively. After excluding the SGA infants from the analyses, the concordance rate of GA-estimation improved to 52%. Exclusion of the SGA infants did not affect the efficacy of weight- and NTL-based estimations.

**Table 3:** Linear regression equations describing the relationships between different parameters to the oro-tracheal distance.

**Parameter**	**Regression equation**	***R* square**
Body weight (kg)	5.9 + 1.1 × body weight	0.76
Ln(Body weight) (kg)	7.1 + 1.7 × Ln(body weight)	0.79
Gestational age (week)	2.5 + 0.2 × gestational age	0.66
NTL (cm)	2.0 + 0.8 × NTL	0.76
Gestational age (week)^*^	2.0 + 0.2 × gestational age	0.78
NTL (cm)^*^	2.1 + 0.8 × NTL	0.78

*Ln, Natural Logarithm; NTL, Nasal Septum to Ear Tragus Length*.

* *Excluding infants small for gestational age*.

**Table 4:** Comparisons of our proposed oro-tracheal intubation depths derived from the different parameters to the recommendations of the Neonatal Resuscitation Program, 7th ed.

**Our study**				**NRP**		
**NTL (cm)**	**BW (gm)**	**GA (week)**	**Suggested intubation length (cm)**	**BW (gm)**	**GA (week)**	**NTL (cm)**
Preterm: +1 cm	≤ 600	≤ 23	6	700–800	25–26	+1 cm
	601–800	24–26	6.5	900–1,000	27–29	
	801–1,200	27–29	7	1,100–1,400	30–32	
	1201–1500	30–32	7.5	1,500–1,800	33–34	
Term: +0.5 cm	1,501–2,200	33–35	8	1,900–2,400	35–36	
	2,201–2,500	36–38	8.5	2,500–3,100	37–38	
	>2,500	39–40	9	3,200–4,200	39–40	

*BW, Body Weight; GA, Gestational Age; NTL, Nasal Septum to Ear Tragus Length; NRP, Neonatal Resuscitation Program*.

## Discussion

This study compared the efficacy of the commonly used intubation length estimation methods for neonatal endotracheal intubation. Our results showed that TF and NTL + 1 cm had similar mid-tracheal concordance, whereas the NRP tables more frequently resulted in shallow endotracheal intubations. NTL + 1 cm provided a more reliable OTD estimation in the EP and ELBW infants than TF and the NRP tables. SGA compromised the efficacy of NTL + 1 cm, but not weight- and GA-based estimations.

Previous studies on the accuracy and efficacy of intubation length estimation methods have reported inconsistent results. Potential sources of these discrepancies include differences in the reference points measured, and the definitions of optimal ETT position and primary outcomes (Table 5). ETT depth is measured from the point of oral fixation to the mid-tracheal tip position. Techniques to secure the ETT vary among NICUs: while some units prefer to tape the ETT at the mouth angle or mid-upper lip, others may use fixation devices to prevent slippage. Although no studies have evaluated the influence of securement methods on OTD, any trivial difference may result in significant ETT displacement within the limited mid-tracheal space in neonates. Previous studies have defined the mid-tracheal position and satisfactory tip position differently, with some defining the mid-trachea as a segment of the trachea, and others as a distinct anatomical point along the trachea. Transforming anatomically defined mid-trachea into radiological landmarks is also clinically challenging. Radiologically, the mid-trachea may be the mid-point between the upper border of T1 and lower border of T2, or the mid-point between the connecting line of inferior clavicles and carina. However, as Beanarek et al. demonstrated in a post-mortem study, the clavicles and T1 are not always aligned (21). Therefore, these different definitions of mid-trachea cannot be considered equivalent. Moreover, some studies did not reposition the ETT if it was not optimally placed in the mid-trachea as long as it was located within predefined satisfactory positions (Table 5). Other studies have required precise estimations of OTDs to place the ETT in a predetermined position, and that even a slight deviation from the measured OTDs was not acceptable. All of these differences could have led to inconsistent conclusions between studies.

Table 5Comparisons of the participants, definition of oro-tracheal distance, and description of satisfactory endotracheal tube position from studies on the different intubation length estimation methods.

**Table:** 

	**Body weight–Tochen's formula**							
	**Tochen (9)**	**Peterson et al. (13)**	**Amarilyo et al. (14)**	**Flinn et al. (15)**	**Gill et al. (16)**	**Chung et al. (17)**	**Tatwavedi et al. (18)**	**Uygur et al. (19)**
Subject, *n*	40	75	31	90	69	139	34	81
GA, mean, wk	32 (26–44)	32 (23–44)	26 (23–30)	29 (27–37)	29 (26–31)	31.2 ± 5.4	31.2 (26–40)	33.4 ± 4.9
BW, mean, gm	1,890 (700–4,100)	2,001 (490–4,400)	762 (407–980)	1,340 (933–2,885)	1,030 (735–1,663)	1713.7 ± 985.9	1,360 (530–2,300)	2,197 ± 1,026
OTD reference measured	Lip to T1 and T2	Upper lip to halfway between inferior clavicle and carina	Mid-upper lip to midway between sterno-clavicular joint and carina	NA	NA	Origin unspecified to halfway between inferior clavicle and carina	Lip to upper border of T2	Midline of mouth to between T1 and T2
Satisfactory tip position	Between T1 and T2	NA	At or close to mid-trachea	Between upper T1 and lower T2	Between upper T1 and lower T2	Between upper T1 and lower T2	Between upper T1 and lower T2	Between T1 and T2
Accuracy, %	95	Average 0.12 cm above mid-trachea	77	55	43	14	71	46

**Table:** 

	**Nasal septum to ear tragus length**			**Gestational age**	
	**Shukla et al**. **(****10****)**	**Uygur et al**. **(****19****)**	**Voraruth et al**. **(****20****)**	**Maine et al**. **(****3****)**	**Flinn et al**. **(****15****)**
Subject, *N*	66	81	110	55	41
Mean GA, wk	31.1 (23–42)	32.5 ± 5.2	NA	NA (23–41)	29 (27–36)
Mean BW, gm	1,610 (955–4,500)	2,009 ± 1,132	NA	NA	1,530 (820–2,830)
OTD reference measured	Angle of mouth to midpoint between clavicles and carina	Midline of mouth to T1 and T2 midpoint	Right mouth angle to T2 body	Lip to a position between T1 and T2	
Satisfactory tip position	Adjustment < 0.5 cm	Between T1 and T2	T2 body	Between T1 and T2	Adjustment < 1 cm
Accuracy, %	95	37	32.7	85	76

*To ensure patient safety and minimize complications, the current consensus is to place the tip of endotracheal tube in the mid-trachea of orally intubated patients. The oro-tracheal distance is the preferred endotracheal intubation depth, and should place the tip of endotracheal tube in the predefined satisfactory positions in proximity to the mid-trachea. However, oro-tracheal distance and satisfactory tip position definitions vary between studies*.

*GA, gestational age; Wk, week; BW, birth weight; OTD, oro-tracheal distance; T1, first thoracic vertebral body; T2, second thoracic vertebral body*.

The rates of correct ETT positioning using TF ranged from 14 to 77% (14, 16, 18). Moreover, the efficacy of TF in predicting the OTD of ELBW infants is often questioned, because the original study had only ten ELBW infants (9). Peterson et al. reported that TF overestimated OTDs in infants under 750 g, but they did not discuss the rates of ETT needed to be repositioned (13). In addition, an Israeli study reported that TF underestimated the OTD in 23% of ELBW infants (14). TF also underestimated the OTD in our participants, however the mid-tracheal concordance rates of TF in the EP and ELBW infants were not inferior to those in the non-EP and non-ELBW infants. In line with previous studies, we found the predicted OTDs using TF were in concordance with the ideal ETT positions in over 50% of our ELBW infants and EP infants (9, 14). The estimated OTDs also did not significantly differ from the measured OTDs in the EP, ELBW, and SGA infants, akin to a recent study which reported that the mid-tracheal concordance of TF was not lowered in SGA infants (17). In contrast, the weight-based NRP recommendations were unreliable, and were concordant with the mid-trachea in only 8.3 and 10.7% of the intubated ELBW and EP infants, respectively. Our revision of the NRP tables improved the mid-tracheal concordance rate by 4-fold using BW, and improved the efficacy of TF by 120%.

In clinical practice, BW is often unavailable and not attainable during post-natal resuscitation. Unlike other external body measurements, NTL can be obtained quickly using an endotracheal tube as a direct measuring tool. The correlation between NTL and OTD was first established by Shukla et al. in 1997 (10). However, physical differences between ethnicities and physiological dehydration after birth can affect morphological measurements. To date, no studies have quantitatively documented such differences in neonates. In this study we examined the efficacy of NTL in Asian neonates, and found that NTL + 1 cm had an overall concordance rate of 57% with the mid-trachea, which is much lower than that originally reported by Shukla et al. (10). We found that NTL significantly overestimated the OTD in term infants, which is consistent with the findings of Wang et al. (22). However, a recent Turkish study reported different results, and found that NTL + 1 cm was more likely to overestimate OTDs in infants <1,500 g and those with a GA < 34 weeks (19). NTL also overestimated OTDs in the SGA infants in the current study. Although SGA did not compromise the overall mid-tracheal concordance of NTL-estimated OTDs, the estimated OTDs were twice as likely to result in intubations that were too deep in the SGA infants. In comparison to the non-SGA infants, NTLs were longer and OTDs were shorter in the SGA infants. The disproportionate increases suggest that NTL is more age-dependent and OTD is more weight-dependent. This also means that age had a greater influence on NTL than BW, which has never been reported in previous studies. The efficacy of NTL + 1 cm in other studies has been reported to be as low as 32.7%, and most of these studies have attempted to refine the efficacy of NTL estimates using BW adjustments (19, 20, 22). Following adjustments for age, there was a 110% improvement in the overall efficacy of NTL estimations and a reduction of over 20% in the rates of too-deep intubations. The efficacy of NTL estimates in the SGA and term infants also improved by 130 and 136%, respectively, although BW-adjustments of NTL estimates did not show such prominent improvements.

The simplified linear relationship between BW and OTD proposed by Tochen et al. has been disputed in later studies (3, 23). Mainie et al. demonstrated a predictable positive correlation between GA and OTD (3). In addition, Kempley et al. tested and refined these preliminary results, and produced a table of recommended ETT lengths by GA, which was later endorsed by the NRP (7, 23). The NRP-GA table was satisfactory in 61% of Kempley's studied subjects. However, an Irish study reported satisfactory ETT positioning in only 39% of patients using the GA-based recommendation (15). The GA-based estimations had an even lower efficacy in our participants, and the estimated OTDs were almost invariably inadequate. We were able to improve the efficacy of GA estimates using the revised intubation table. However, despite the substantial improvement in concordance from 15% to 44%, the efficacy of GA-based estimates was still disappointing compared to those of NTL and weight-based estimations. The lower efficacy of GA-based estimations agreed with our assumption that BW has a greater influence on OTDs than GA.

Table 4 compares our revisions to the NRP recommendations. In general, the OTD was deeper in the Taiwanese infants with a birth weight <1,500 g and in those <35 weeks of gestation, while OTD was shorter in the term Taiwanese infants. Taiwanese preterm infants have been reported to have a heavier average weight compared to infants of other ethnicities, with this disparity being sustained until late preterm gestation (12, 24). However, BW has been reported to be a poor indicator of tracheal length in pediatric and adult populations, with body height being more predictive of lower airway dimensions (25). Further studies are needed to elucidate the influences of various anthropometric measurements on OTDs.

The limitation of this study is the lack of information regarding radiological and clinical complications secondary to unsatisfactory ETT positioning. This information could have heightened and reiterated the importance of effective and precise endotracheal intubation in neonates. An intrinsic limitation of OTD measurements is the absence of detailed gradation on an endotracheal tube precluding accurate fixation to the nearest decimal place. The recommendations were revised based on Taiwanese preterm infants. The inclusion of a large number of ELBW and EP infants is both a limitation and strength of the study. Because 22% of the participants were term infants and only 16% were non-low birth weight (BW > 2,500 g), future studies are needed to validate the recommended intubation lengths in these infants. Nonetheless, the composition of the participants in this study is more representative of a modern NICU patient population compared with previous studies, and our results may help to prevent avoidable complications in vulnerable ELBW and EP infants. We performed direct comparisons of the efficacy of the four different methods commonly practiced and endorsed by the NRP.

## Conclusions

NTL + 1 cm had better mid-tracheal concordance than TF and the NRP tables. In the ELBW and EP infants, TF was more likely to overestimate the OTD, while NRP intubation tables were more likely to underestimate the OTD in Taiwanese infants. In SGA infants, measured OTDs were more strongly correlated with weight-based estimations than GA-based estimations. Our revised NRP intubation table based on our participants improved the efficacy of BW, NTL, and GA-derived OTD estimations. Future studies are needed to validate our results in different settings and other populations.

## Data Availability Statement

The raw data supporting the conclusions of this article will be made available by the authors, without undue reservation.

## Ethics Statement

The studies involving human participants were reviewed and approved by MacKay Memorial Hospital. Written informed consent to participate in this study was provided by the participants' legal guardian/next of kin.

## Author Contributions

H-YC, C-HH, J-HC, W-TJ, and C-YL contributed to the conception and design of the study. C-HC, N-PT, and MK organized the database. MK and C-HC performed the statistical analysis. C-CP and MK wrote the first draft of the manuscript. N-PT, C-CP, and MK wrote the sections of the manuscript. All authors contributed to the article and approved the submitted version.

## Conflict of Interest

The authors declare that the research was conducted in the absence of any commercial or financial relationships that could be construed as a potential conflict of interest.

## Abbreviations

BW: body weight
CXR: chest roentgenogram
ELBW: extremely low birth weight (birth weight ≤ 1,000 g)
EP: extremely preterm (gestational age ≤ 28 weeks)
ET: endotracheal tube
ETT: endotracheal tube tip
GA: gestational age
NICU: neonatal intensive care unit
NRP: neonatal resuscitation program
NTL: nasal septum to ear tragus length
OTD: oro-tracheal distance
SGA: small for gestational age
T1: first thoracic vertebral body
T2: second thoracic vertebral body
T1T2: between the upper margin of the first thoracic vertebral body to the lower margin of the second thoracic vertebral body
TF: Tochen's formula.
